# Mechanocardiography in the Detection of Acute ST Elevation Myocardial Infarction: The MECHANO-STEMI Study

**DOI:** 10.3390/s22124384

**Published:** 2022-06-09

**Authors:** Tero Koivisto, Olli Lahdenoja, Tero Hurnanen, Tuija Vasankari, Samuli Jaakkola, Tuomas Kiviniemi, K. E. Juhani Airaksinen

**Affiliations:** 1 Department of Computing, University of Turku, Vesilinnantie 5, 20500 Turku, Finland; tejuko@utu.fi (T.K.); tero.hurnanen@utu.fi (T.H.); 2 Heart Center, Turku University Hospital, Hämeentie 11, 20520 Turku, Finland; tuija.vasankari@tyks.fi (T.V.); samuli.jaakkola@tyks.fi (S.J.); tuomas.kiviniemi@tyks.fi (T.K.); juhani.airaksinen@tyks.fi (K.E.J.A.)

**Keywords:** accelerometer, gyroscope, seismocardiography, telemonitoring, acute myocardial infarction, STEMI, electrocardiography, ECG

## Abstract

Novel means to minimize treatment delays in patients with ST elevation myocardial infarction (STEMI) are needed. Using an accelerometer and gyroscope on the chest yield mechanocardiographic (MCG) data. We investigated whether STEMI causes changes in MCG signals which could help to detect STEMI. The study group consisted of 41 STEMI patients and 49 control patients referred for elective coronary angiography and having normal left ventricular function and no valvular heart disease or arrhythmia. MCG signals were recorded on the upper sternum in supine position upon arrival to the catheterization laboratory. In this study, we used a dedicated wearable sensor equipped with 3-axis accelerometer, 3-axis gyroscope and 1-lead ECG in order to facilitate the detection of STEMI in a clinically meaningful way. A supervised machine learning approach was used. Stability of beat morphology, signal strength, maximum amplitude and its timing were calculated in six axes from each window with varying band-pass filters in 2–90 Hz range. In total, 613 features were investigated. Using logistic regression classifier and leave-one-person-out cross validation we obtained a sensitivity of 73.9%, specificity of 85.7% and AUC of 0.857 (SD = 0.005) using 150 best features. As a result, mechanical signals recorded on the upper chest wall with the accelerometers and gyroscopes differ significantly between STEMI patients and stable patients with normal left ventricular function. Future research will show whether MCG can be used for the early screening of STEMI.

## 1. Introduction

Most deaths caused by acute ST elevation myocardial infarction (STEMI) occur out of hospital within the first hour after symptom onset [1]. No significant improvement in the prehospital delay has occurred during the last decade and, strikingly, every other STEMI patient waits at least 2 h after symptom onset before seeking medical help [2]. Minimizing these delays is an important target to improve the prognosis of STEMI.

Mechanocardiography (MCG), including seismocardiography and gyrocardiography, records the micro-vibrations generated rhythmically as a consequence of the movements of cardiac mass and blood in the major vessels, respectively, with micro-accelerometers and gyroscopes placed on the body surface [3,4,5]. We have earlier shown that a smartphone application using MCG is a clinically useful methodology for the screening of atrial fibrillation and yields better diagnostic accuracy than currently available wearable devices using photoplethysmography signal [6]. Previous research has demonstrated that waveforms derived from seismocardiography and gyrocardiography reproduce the main events seen in left ventricular twist and strain rates obtained from echocardiography [3,7]. Experimental evidence also suggests that abnormalities in the heart function caused by myocardial infarction could be detected using this technology [8,9]. Clinical data on these techniques is still limited, but wearable seismocardiography can help to assess the clinical status of patients with heart failure using a machine learning algorithm [10]. In this proof-of-concept study, we use a supervised machine learning pipeline that consists of the development of novel features and feature selection combined with logistic regression classifier with leave-one-person-out cross-validation to investigate whether the information provided by MCG facilitates detection of abnormalities in cardiac function caused by STEMI.

The concept that MCG could be utilized to detect STEMI has been initially investigated by us in [11] using smartphones. In this paper, however, a dedicated monitoring solution using a custom single-lead holter monitor equipped with MCG sensing is utilized. The reason for this is that joint ECG allows better localization of features (enabled by R-peak from single lead ECG), allowing the construction of feature set which is not only discriminative but can also be interpreted meaningfully by cardiologists. This is essential in order to understand the overall system functionality. In comparison with the previous studies (e.g., [9,11]) also a more comprehensive study group is used in combination with advanced automated motion artefact cancellation technique.

## 2. Background and Motivation

### 2.1. Background

The gold standard for the detection of STEMI is 12-lead ECG. In the past, several algorithms have been developed for the automated detection of STEMI using 12 (or less)-lead ECG. In [12], an artificial neural network (ANN) method was shown to outperform a rule-based method in classifying myocardial infarction patients using 12-lead ECG. Bayesian ANN and Hermite basis functions were used in [13] to detect STEMI in using both 12-lead and 8-lead ECG configurations. In general, adding more leads tends to yield better classification accuracy. In [14], ensemble ANN was compared to multiple logistic regression classifier using 12-lead ECG. If experienced cardiologists are not available, automated algorithms can also be used assist doctors in making the correct diagnosis based on 12-lead ECG [15]. In these settings, the threshold of the classifier (which generally determines how many false positives or false negatives are allowed) is tuned to eliminate potential of classifying STEMI as non-STEMI. Recently, in [16], Fourier–Bessel series combined with deep neural network (DNN) was used to classify myocardial infarction using 12-lead ECG with very high accuracy.

Currently, applying 12-lead ECG for STEMI detection in ambulatory settings is difficult since it is not ubiquitously available. Using fewer leads to enable better portability is a viable option [17], but generally also reduces the detection accuracy of STEMI. Either false positive or, more significantly, false negative results may be introduced. In mobile settings, the detection can work such that the patient triggers the analysis themselves, e.g., when suspecting chest pain symptoms, or continuous-time monitoring is performed automatically either locally or by sending the data to a cloud through Internet-of-Things (IoT). In order to cope with the portability issues, it has been proposed that multiple serial measurements could be performed with single-lead ECG in different chest locations to enable multi-lead ECG generation [18]. However, this comes with a trade-off, as the overall ECG acquisition time becomes then longer. An implantable device is also an option [19] as it enables continuous time monitoring without portability issues, but on the other hand it can only be considered for limited number of risk group patients due to potential surgical complications.

### 2.2. Motivation

The majority of fatalities due to STEMI occur in low-risk coronary patients or in “healthy” subjects without any history of cardiac disease [20,21,22,23,24]. The reason for this is that the patient often stays home to follow the symptoms or does not recognize them, meaning the start of treatment is delayed. Sudden coronary occlusion at the site of a vulnerable plaque with only mild stenosis, not serious enough to rise to chronic chest pain symptoms, may trigger fatal ventricular fibrillation or cardiac asystole in these patients. Vulnerable plaques occur across the full spectrum of severity of coronary stenosis. Advanced therapies such as coronary angioplasty or implantable defibrillators are of no value to the majority of victims, who do not survive to reach the medical care.

The peak hazard rate for primary ventricular fibrillation occurs at 25–30 min from the onset of symptoms while the median delay between onset of symptoms of ST elevation myocardial infarction and seeking medical attendance range from 1 to 4.5 h. The time it takes for the person to decide how to interpret and respond to symptoms is considered to be the main contributor to prehospital delay, which has not improved for decades. For example, over 50% of myocardial infarction patients wait at least 2 h after symptom onset before arriving at hospital [25] (e.g., due to potential embarrassment caused by a false alarm).

**Figure 1:**
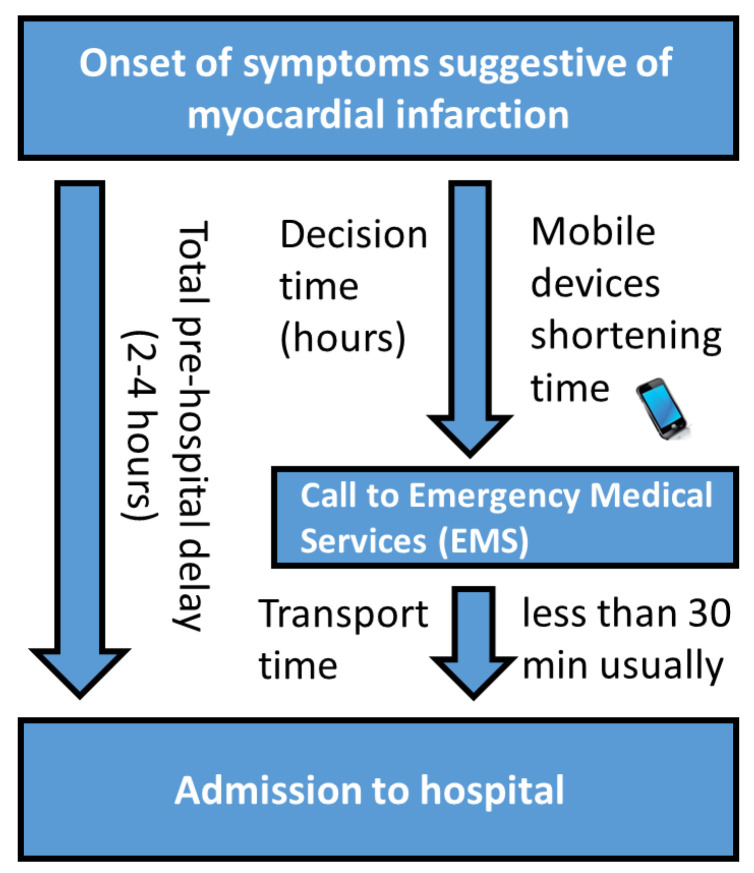
Total pre-hospital delay.

At present, the prognosis of patients with acute myocardial infarction reaching the hospital for primary coronary angioplasty is good and the hospital mortality rate is <2% in middle-aged patients. Thus, the major challenge in the treatment of acute myocardial infarction is to minimize the unnecessary patient-related delays in seeking for medical care after the emergence of symptoms suggestive of acute coronary syndrome (see Figure 1).

## 3. Materials and Methods

### 3.1. Patients

MECHANO-STEMI study (Mechanocardiography in patients with STEMI; ClinicalTrials.gov Identifier: NCT03441724) is a single-center prospective study to assess the potential of MCG in the screening for cardiac mechanical function abnormalities caused by STEMI. The study protocol was approved by the Ethics Committee of the Hospital District of Southwest Finland, and the study was performed in accordance with the Declaration of Helsinki as revised in 2002. All patients gave their written informed consent.

In this study, initially MCG recordings were performed in 60 patients admitted with acute STEMI and 69 control patients referred for elective coronary angiography because of suspected stable coronary artery disease. Patients with significant valve disease or rhythm irregularities were excluded and all control patients had normal left ventricular ejection fraction in echocardiography. From this group, 19 STEMI patients and 20 control patients were excluded due to rhythm irregularities and/or poor ECG quality for automated R peak detection. Thus, 41 patients with STEMI and 49 control patients formed the final study groups (Table 1). Comparisons between groups in Table 1 were performed with the Chi-square test for categorical variables and Mann–Whitney U-test for analysis of continuous data. Statistical analyses were performed using version R2017a of Matlab and Python 3.8.3.

According to Table 1 STEMI patients had slightly higher heart rate and lower systolic blood pressure. In the STEMI group, the median delay from the pain onset to the recording was 139 min (range 10–690 min). The mean of troponin T peak values was 4189 ng/L. The culprit lesion causing STEMI was in left anterior descending artery in 19 patients, in right coronary artery in 14 patients, in left circumflex artery in 6, and in diagonal branch in 2 patients.

### 3.2. Data Acquisition

**Figure 2:**
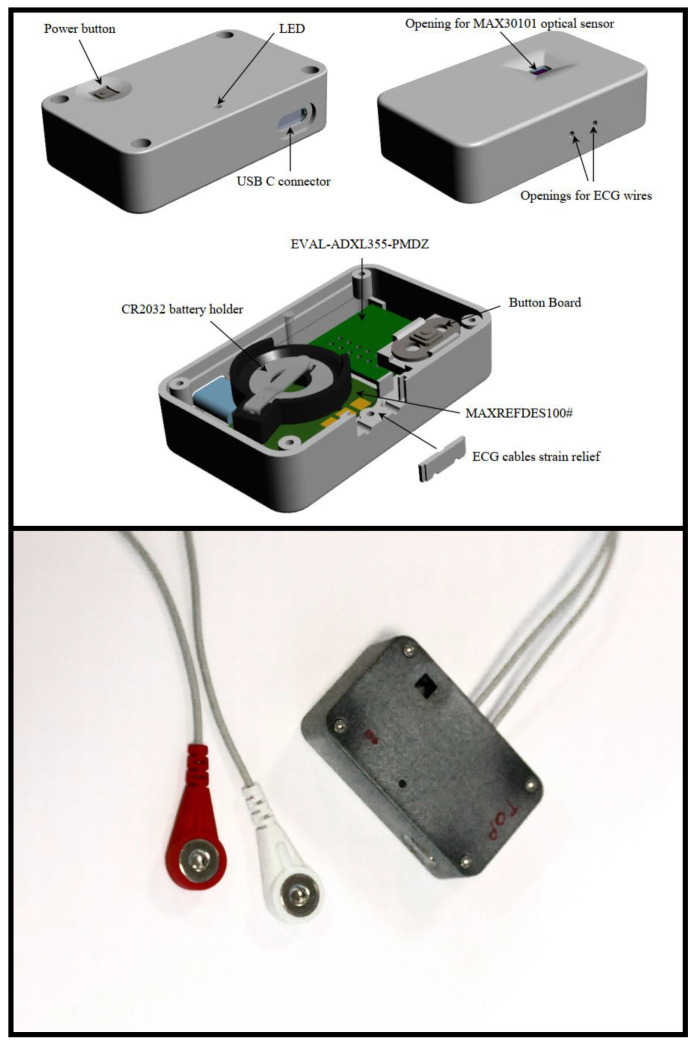
Device used in data acquisition including 3-axis accelerometer, 3-axis gyroscope and single lead ECG for R-peak extraction. The optical sensor is not used in this study.

All recordings were performed in supine position upon arrival to the catheterization laboratory using a custom-designed data logger equipped with a 3-axis accelerometer (ADXL355), 3-axis gyroscope (LSM6DS3) and single-lead ECG (Figure 2). The data logger was based on a Maxim MAXREFDES100# health sensor (hsensor) platform which was modified in order to enable operation of an ADXL355. The data logger was attached to the skin in the upper sternum using double-sided tape without hair removal. The measured acceleration and angular velocity range of the accelerometer and gyroscope were set to ±2 g and ±250 dps, respectively. The accelerometer had a noise density of 25 μg/√Hz while the gyroscope rate noise density was 7 mdps/√Hz. All measurements were processed using a custom-made software. All MCG and ECG data were recorded simultaneously and resampled to have a sampling frequency (Fs) of 200 Hz. A 4th-order Butterworth IIR filter with pass bands 1–90 Hz, respectively, were applied on the gyroscope- and accelerometer-derived signals, allowing the removal of white noise, signals offset and respiration.

### 3.3. Motion Artefact Removal

An initial length of the signals was up to 900 s (mean 864.4 s). Motion artefacts affect seismocardiographic and gyrocardiographic waveforms more than ECG and a customized artefact-removal algorithm was applied prior to signal filtering. In short, the algorithm divides the signal into short non-overlapping segments of 5 s and goes through each segment using a 1 s window. If any of these windows exceeds a pre-specified noise threshold limit in any axis, the segment was rejected. The noise estimate was calculated based on signal traversal length (cumulative value of absolute signal change within window). Finally, the longest consecutive (no gaps between beats) segment was used as an artefact-free signal for the analyses. Mean length of artefact-free signal was 234.6 (SD = 157.6) seconds in the STEMI group and 221.3 (SD = 109.6) seconds in the control group. The same noise thresholds were applied for all measurements.

### 3.4. ML Pipeline

**Figure 3:**
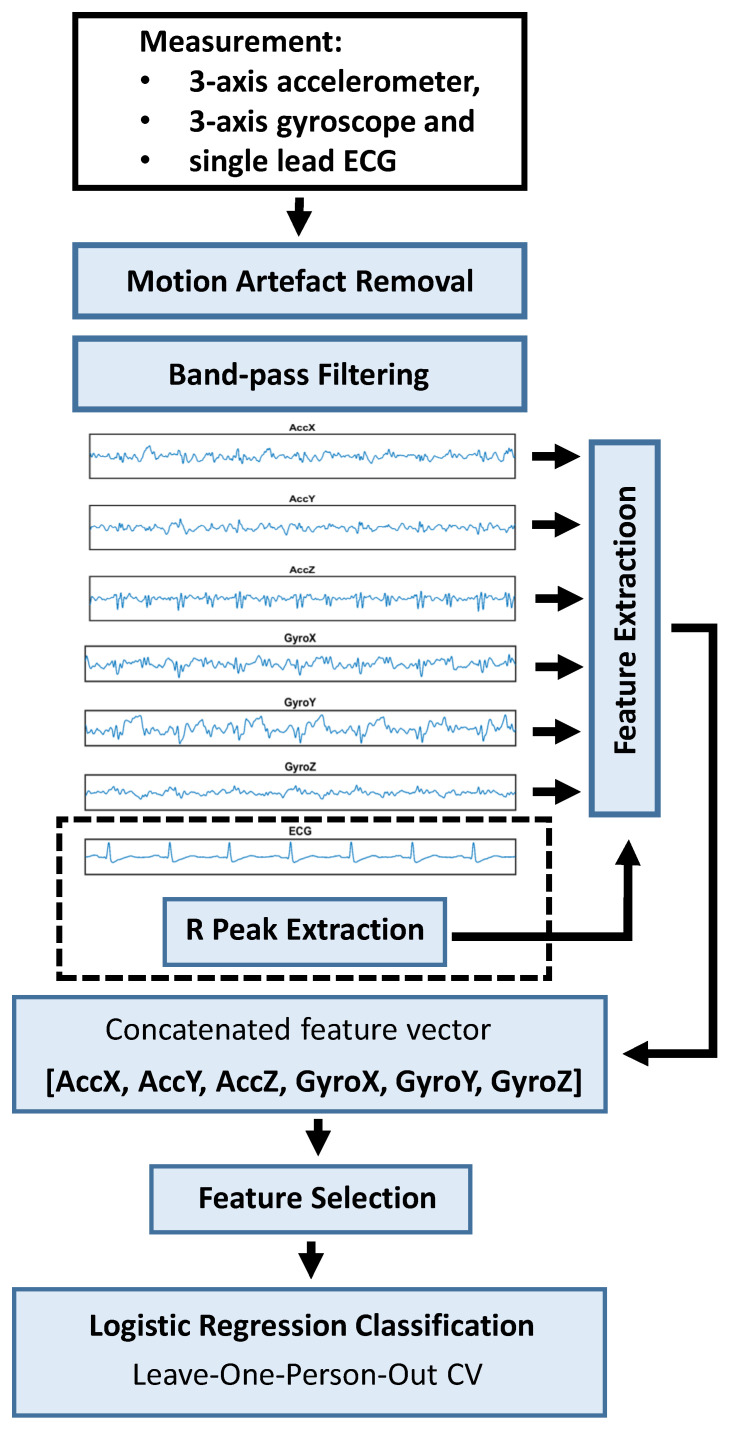
The overall measurement and analysis pipeline. First motion artefact removal selects only the longest connected artefact-free signal portion (in all axes). Then resulting signal was band-pass filtered. ECG R peaks were used to locate heartbeats for feature extraction and three main feature types were analyzed: stability of heartbeat morphology, signal strength and amplitude and time intervals (calculated within specific windows inside each heartbeat and axis). Finally, feature selection and binary classification with logistic regression classifier utilizing cross validation (LOOCV) were performed.

We used a supervised machine learning (ML) pipeline which contains the steps of extracting a set of descriptive features from the measurement signals followed by feature selection and classification utilizing cross-validation (Figure 3). All the experiments were run on Dell Latitude Laptop PC equipped with Intel Core i5 and Matlab R2017a (MathWorks, Inc., Natick, MA, USA). The automatically detected R-peaks were used to locate systole and diastole together with location of heart sounds based on typical mechanical (i.e., accelerometer and gyroscope) signals. Features were usually extracted from all six axes, except for certain signal amplitude and timing features which utilized only AccZ axis (Earth gravity direction while patient is in supine position).

The features from each 6 MCG axes were concatenated to a final feature vector in the following way. First, the feature vector extracted from AccX axis was placed to the beginning of the final feature vector, then AccY axis features and finally GyroZ axis features. The last feature of GyroZ axis is then also the last feature of the concatenated feature vector, and the first feature of AccX axis the first feature. Thus, if there were N features in each axis, the final feature vector length would become 6 (the number of MCG axes) times N (Figure 3). Three main types of features were used.

#### 3.4.1. Feature Extraction

**Beat stability features:** The first of these was the inter-beat stability between windows of duration between 0.25 and 0.75 s, centered at systole, diastole and heart sounds. Multiple features corresponding to each window were extracted from each signal axis by band-pass filtering the signal with an upper frequency of 90 Hz and lower frequencies of 2, 20, 30 and 40 Hz. The content of each window within a specific signal axis was compared using a Pearson correlation to all other windows within the same axis from fixed number of beats. Thus the measured “beat stability” is higher when the correlation is larger and vice versa. From the extracted correlation matrix both mean and median values were calculated. In total there were 240 stability features.

**Signal strength features:** Overall and window (i.e., windows centered at systole, diastole and heart sounds) specific signal strengths were calculated in each axis using average root mean square value of the signal within each window. Additionally, relative signal strengths (e.g., systole/diastole) were calculated within each axis. In total, there were 252 signal strength features.

**Signal amplitude and timing features:** Mean and median of maximum signal amplitudes at specific locations, e.g., around third heart sound inside a window—in contrast to total signal strength average within the window—in each axis (e.g., AccZ) were calculated together with standard deviations of the feature values. In total there were 121 timing and amplitude features.

#### 3.4.2. Feature Selection and Classification

Feature selection was based on group comparisons with Mann–Whitney U test. The signal features with 150 best *p*-values in an ascending order were used for primary classification. We also report the result with all 613 features, which include all beat stability, signal strength and signal amplitude and timing features.

Different classifiers (i.e., linear support vector machine—SVM, random forest—RF, logistic regression—LR) were tested during the development and LR was chosen based on best performance. Leave-one-person-out cross validation (LOOCV) was used to validate the performance of the classifier. In LOOCV, one person at a time is always left out from the training set and the classifier is then trained with the features from all remaining (a total of 89) patients. The class of the patient which was left out is then (independently) evaluated using the classifier trained on the training set. This is iterated as many times as there are patients (i.e., feature vectors, i.e., total of 90 times). Multiple LOOCV iterations (a total of 10) were used with different random seeds. To evaluate the performance of the classifiers, the area under the receiver operating characteristic curve (AUC) was calculated in the prediction of STEMI.

## 4. Results

### 4.1. Classification Results

**Figure 4:**
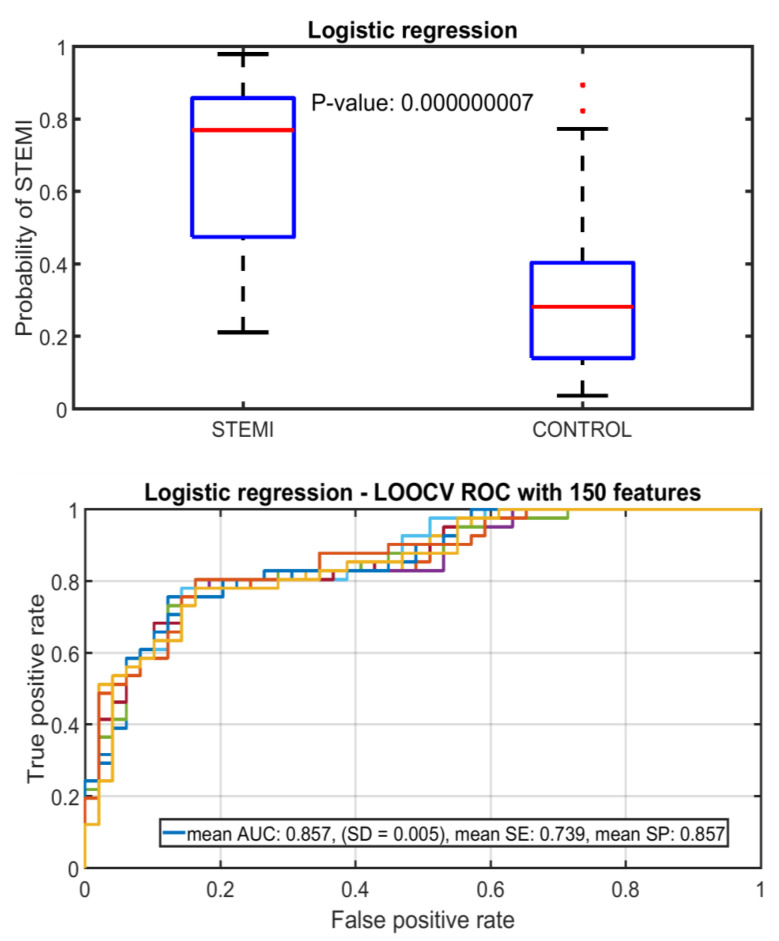
Box plot and receiver operating characteristic (ROC) curve with 150 best features using logistic regression classifier to separate STEMI and control patients.

In logistic regression analysis using the 150 best signal features, the difference in MCG findings between STEMI and control patients was statistically highly significant (*p* = 0.000000007) (Figure 4). Using logistic regression classifier and leave-one-person-out cross validation, a sensitivity of 73.9%, specificity of 85.7% and AUC of 0.857 (SD = 0.005) was obtained using the 150 best features. The mean accuracy was 80.3%. Including all features (a total of 613) had only a minimal effect on classification and AUC was 0.861 (SD = 0.006).

Comparison to the state-of-the-art is shown in Table 2. The modalities include 1-lead ECG (using serial measurements) [18], 3-lead ECG (RELF system) [17], 8-lead ECG [13] and 12-lead ECG [12,13,14,15,16]. Observe that the methods are very different in terms of enabling patient mobility and this directly affects the accuracy also. Thus, each method have their advantages and disadvantages. Our method (assuming 1-lead ECG would not be needed in the future) is not only wearable but also ubiquitously available. This allows using the approach for patients other than those in the risk group. RELF system [17] is operated in continuous time and targeted for patients in the risk group only. However, for these patients, the accuracy of the method is very good. Moreover, different methods generally utilize different recording lengths. Although the method in [16] utilizing 12-lead ECG shows the highest accuracy, it must be noted that it also uses full 24 h recordings, whereas the method in [18] uses signals of less than 6 min duration. Invasive methods were not included in this table.

### 4.2. Feature Analysis

**Figure 5:**
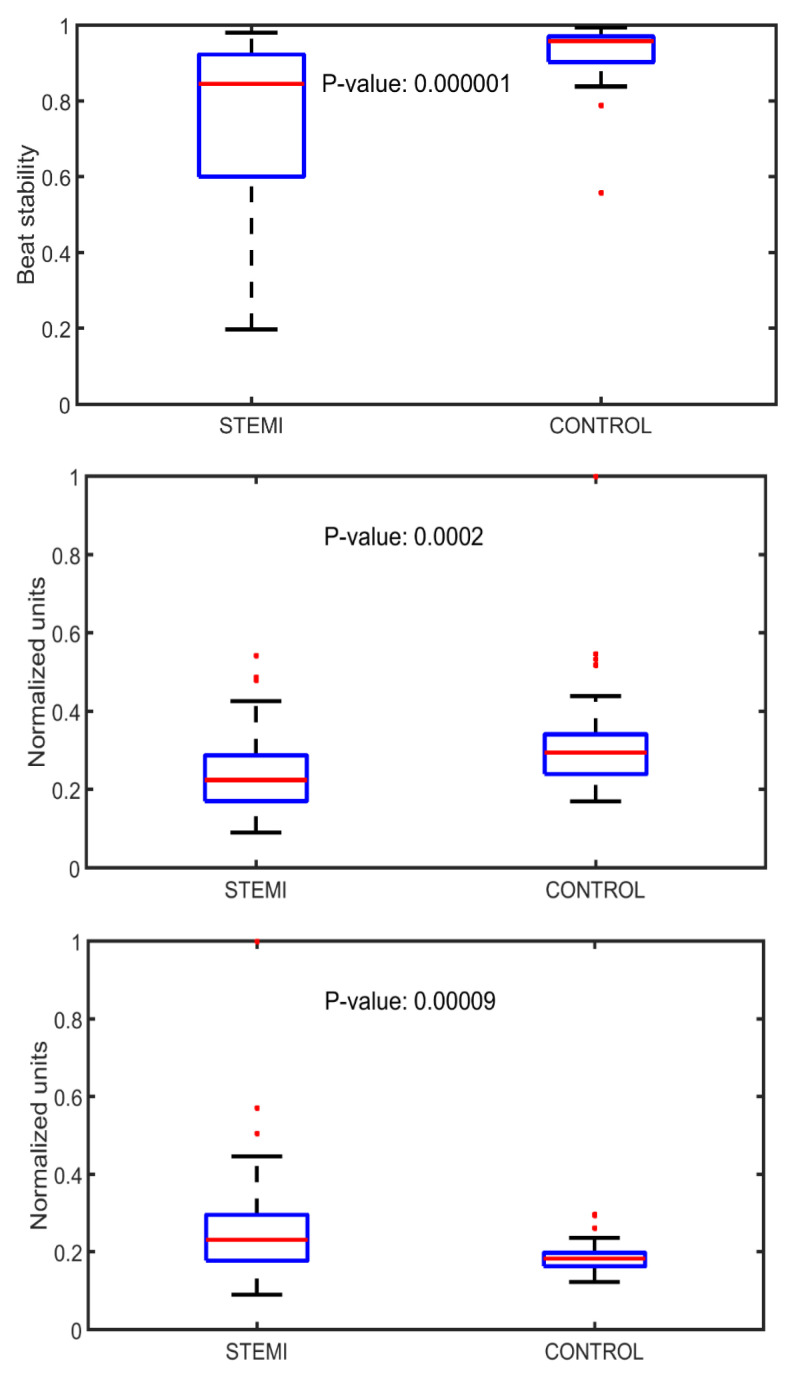
Plots representing median of beat stability (band-pass filter 20–90 Hz) in AccX axis in a window around second heart sound (**Upper** panel), systole/diastole ratio in signal strength in GyroX axis (**Middle** panel), and median value of signal amplitude (band-pass filter 10–80 Hz) around third heart sound in AccZ axis (**Lower** panel) in STEMI and control patients.

The stability features of waveform structure gave consistently lower values among STEMI patients within all axes and window locations. The most prominent difference (*p* = 0.000001) was seen when using a window around the second heart sound (Figure 5) and when using 120 best stability features, an area under curve (AUC) of 0.829 was obtained.

**Table 2:** Comparison of automated STEMI detection methods.

Study	Modality	Wearable	Method	Patients	Duration of	Sensitivity	Specificity
		**Device**		**(STEMI/Control)**	**Recordings**		
Heden et al., (1997) [12]	12-lead ECG	No	ANN	1120/10,452	NA	46.2%	95.4%
Heden et al., (1997) [12]	12-lead ECG	No	Rule-based	1120/10,452	NA	30.7%	95.4%
Haraldsson et al., (2004) [13]	12-lead ECG	No	Bayesian ANN	1119/1119	NA	63.3%	85.0%
Haraldsson et al., (2004) [13]	12-lead ECG	No	Hermite functions	1119/1119	NA	61.5%	85.0%
Haraldsson et al., (2004) [13]	8-lead ECG	No	Bayesian ANN	1119/1119	NA	56.3%	85.0%
Haraldsson et al., (2004) [13]	8-lead ECG	No	Hermite functions	1119/1119	NA	59.3%	85.0%
Green et al., (2006) [14]	12-lead ECG	No	ANN ensemble	130/504	NA	95.0%	41.4%
Green et al., (2006) [14]	12-lead ECG	No	Logistic regression	130/504	NA	95.0%	33.7%
Olsson et al., (2006) [15] *	12-lead ECG	No	Feed-forward ANN	736/3264	NA	95.0%	88.0%
Tripathy et al., (2019) [16]	12-lead ECG	No	Fourier-Bessel DNN	100/52	24 h	99.9%	99.6%
Van Heuverswyn et al., (2019) [17]	3-lead ECG	Yes	Rule-based (ST-seg.)	59 (5011 rec.)	NA	87–100%	96.0%
Spaccarotella et al., (2020) [18]	1-lead ECG x 9	Yes	Human expert	54/19	5.8 min	93.0%	95%
Ours **	MCG (1-lead ECG)	Yes	Logistic regression	41/49	15 min	85.7%	73.9%

* transmural ischemia only, ** sinus rhythm recordings only.

Several significant differences between STEMI patients and controls were observed also in signal strength. Generally, the strength features in systolic windows were lower in STEMI patients than in controls. Statistically, the most significant difference was observed in systole/diastole signal strength ratio in the GyroX axis which was significantly (*p* = 0.0002) lower in STEMI patients (Figure 5).

Regarding timing and amplitude features, the most significant difference was observed in the median value of the maximum amplitude around the third heart sound (S3) in the AccZ axis, which was higher (*p* = 0.00009) in STEMI patients. The additive effect of timing and amplitude features on patient classification was relatively small.

## 5. Discussion

This study, using a supervised machine learning approach, showed that the MCG waveforms recorded on the upper chest wall differ significantly in patients with acute STEMI compared to patients undergoing elective coronary angiography or intervention. The most significant differences were the lower waveform stability in the window around second heart sound and lower systole/diastole signal strength ratio in STEMI patients. Similar changes (in the same direction) were observed among other features (i.e., with varying band-pass ranges and window sizes) also.

Seismocardiography measures the vibrations of the chest wall in response to the movement of the heart in the chest, whereas ballistocardiography measures the overall body motion caused by movement of blood through the arterial tree [26,27]. The seismocardiogram consists of waves in the time domain that correspond to aortic valve opening and closing events, as well as the rapid ejection of blood into the aorta. Unlike impedance-based measurements, seismocardiogram is not confounded by fluid shifts in the body and does not require the application of a large number of electrodes on the chest. Gyrocardiography with a 3-axis gyroscope on the patient’s chest has a potential to reveal some of the rotational aspect of heart movement. Growing evidence has shown that gyrocardiography and seismocardiography provide information on the cardiac contractile status, showing a correlation with stroke volume and myocardial contractility [5,26,27,28].

Previous experimental studies have shown significant waveform changes during acute myocardial infarction with 3-axes linear micro-accelerometers, suggesting that this methodology might be useful in the early detection of STEMI also in the clinical setting [8,29,30]. Most recently, it was demonstrated that using multi-dimensional seismocardiography, a significant drop in cardiac kinetic energy during experimental transmural myocardial infarction reflected a decrease in left ventricular contractile function [8]. In accordance with earlier experimental studies, we could observe a weakening of MCG signal strength in systolic windows in STEMI patients compared with control patients having normal left ventricular function.

Phonocardiography has shown progressive development of S3 together with a decrease in the amplitude of S1 during acute myocardial infarction [31]. Amplitude of S3—including nonaudible components—can be measured with accelerometers [32] and exaggeration of S3 is a well-known feature of left ventricular dysfunction and elevated filling pressure [33]. In line with this background, the amplitude of S3 was higher in STEMI patients. The inter-individual scatter in all signal features and their combination was, however, large in both the STEMI and control groups, with significant overlap between the groups, and the causes of misclassification could not be identified.

In a final solution, instead of using 1-1 misclassification cost between the classes it could be feasible to consider increasing the misclassification cost for the STEMI class. Increasing the sensitivity would, on the other hand, decrease specificity and increase the number of false alarms. Moreover, our dataset can be used to examine the effect of ruling out of STEMI by estimating specificity when sensitivity becomes 100%. According to the ROC curve (in Figure 4), it is at approximately 40% specificity that all the STEMIs become correctly classified. However, the overall rate of false positives/false alarms tolerated by a final telemonitoring solution depends on many issues. In general, before attempting to use any system towards minimizing the delay for care-seeking during STEMI in real use, more extensive blinded clinical validations and risk analyses will be required, together with certifying the overall system functionality.

The approaches in Table 2 were different in terms of test setup and targeted usage scenarios, and comparing the performance of the approaches should not be based on detection accuracy only. Each method has their own advantages and disadvantages. Instead of comparing the detection rates as such, the purpose of the comparison was to highlight the different trade-offs between the methods, such as between the detection accuracy and potential scalability or availability of the method for wider use in the future. Our methodology has obvious limitations that need to be addressed. The accuracy of this noninvasive approach may be affected by individual variations in the anatomy and orientation of heart, and thoracic aorta together with marked differences in body mass distribution, which may affect MCG signals recorded on the chest wall. A variable increase in sympathetic activity may also affect cardiac kinetic energy computed from seismocardiography and gyrocardiography [34].

The limited number of patients is a major limitation, together with a selected control group with normal left ventricular and valve function and heart rhythm. Due to small patient groups, it was not feasible to divide the patients into training and test sets before cross-validation. In addition, STEMI patients suffer from severe pain and anxiety, which may have nonspecific effects on MCG findings and signal quality and the initial length of MCG recordings was long (i.e., 15 min) for clinical purposes. To minimize the effects of mental stress we, however, selected the control group from among patients referred for elective coronary angiography and coronary angioplasty, and all recordings were performed in the same environment at a catheterization laboratory.

Future research will show whether a better separation can be reached using self-similarity analysis, i.e., each subject has their individual MCG recording patterns in normal conditions and during STEMI. Due to the ubiquitous availability of devices equipped with MCG sensors, another future research direction could be to assess whether the combination of MCG and e.g., ST-segment analysis based on single-lead ECG would improve the overall accuracy in comparison with single modality only.

## 6. Conclusions

Our findings show that the mechanical signals recorded on the upper chest wall with the accelerometers and gyroscopes differ significantly between STEMI patients and stable patients with normal left ventricular function. Most STEMI patients could be separated from the controls, but so far the accuracy of the present approach seems insufficient for clinical decision making. Thus, prior to even attempting to use this technology in any real use scenario, more extensive and blinded clinical validations and risk analyses together with certifying the overall system functionality are required. In the future research, we will examine whether better separation can be reached using serial measurement analysis where each subject has their individual MCG recording patterns in normal conditions and during STEMI. The final goal is to assess whether this technology being included, e.g., in modern smartphones could help patients with chest discomfort in self-evaluation for STEMI and shorten the delays in care-seeking during a heart attack.

## Figures and Tables

**Table 1:** Demographics.

	STEMI Patients	Control Patients	*p*-Value
	***n*** **= 41**	***n*** **= 49**	
Male	25 (61.0)	39 (79.6)	0.088
Weight, kg	80.1 ± 18.6	84.8 ± 14.8	0.198
Height, cm	174.0 ± 10.5	174.1 ± 9.4	0.888
Age, years	66.8 ± 13.8	65.9 ± 10.3	0.874
Syst. blood pressure, mmHg	129.6 ± 27.2	140.1 ± 20.5	0.037
Diast. blood pressure, mmHg	71.3 ± 15.9	72.2 ± 10.7	0.401
Heart rate, bpm	77.3 ± 22.4	65.8 ± 10.8	0.026
Maximum troponin T, ng/L	4189 (5827)	N/A	-
Ejection fraction, mean,%	37.8 ± 12.8	60.2 ± 7.3	<0.001
Body mass index, kg/m^2^	26.1 ± 4.5	28.0 ± 3.8	0.164
Beta blocker	11 (26.8)	16 (32.7)	0.712
Coronary artery disease	39 (95.1)	39 (79.6)	0.065

Data presented as mean ± standard deviation or count (%).

## Data Availability

The data used in the study is governed by Finnish law of confidential clinical data and will not therefore be made available publicly.

## References

[1] Salomaa V., Ketonen M., Koukkunen H., Immonen-Räihä P., Jerkkola T., Kärjä-Koskenkari P., Mähönen M., Niemelä M., Kuulasmaa K., Palomäki P. (2003). Decline in out-of-hospital coronary heart disease deaths has contributed the main part to the overall decline in coronary heart disease mortality rates among persons 35 to 64 years of age in Finland: The FINAMI Study. Circulation.

[2] Karam N., Bataille S., Marijon E., Tafflet M., Benamer H., Caussin C., Garot P., Juliard J., Pires V., Boche T. (2019). Incidence, mortality, and outcome-predictors of sudden cardiac arrest complicating myocardial infarction prior to hospital admission. Circ. Cardiovasc. Interv..

[3] Tadi M.J., Lehtonen E., Saraste A., Tuominen J., Koskinen J., Teräs M., Airaksinen J., Pänkäälä M., Koivisto T. (2017). Gyrocardiography: A new non-invasive monitoring method for the assessment of cardiac mechanics and the estimation of hemodynamic variables. Sci. Rep..

[4] Gurev V., Tavakolian K., Constantino J., Kaminska B., Blaber A.P., Trayanova N.A. (2012). Mechanisms underlying isovolumic contraction and ejection peaks in seismocardiogram morphology. J. Med. Biol. Eng..

[5] Sieciński S., Kostka P.S., Tkacz E.J. (2020). Gyrocardiography: A review of the definition, history, waveform description, and applications. Sensors.

[6] Jaakkola J., Jaakkola S., Lahdenoja O., Hurnanen T., Koivisto T., Pänkäälä M., Knuutila T., Kiviniemi T.O., Vasankari T., Airaksinen K.E.J. (2018). Mobile phone detection of atrial fibrillation with mechanocardiography: The MODE-AF Study. Circulation.

[7] Morra S., Hossein A., Rabineau J., Gorlier D., Racape J., van de Borne P. (2021). Assessment of left ventricular twist by 3D ballistocardiography and seismocardiography compared with 2D STI echocardiography in a context of enhanced inotropism in healthy subjects. Sci. Rep..

[8] Morra S., Pitisci L., Su F., Hossein A., Rabineau J., Racape J., Gorlier D., Herpain A., Migeotte P., Creteur J. (2021). Quantification of Cardiac Kinetic Energy and Its Changes During Transmural Myocardial Infarction Assessed by Multi-Dimensional Seismocardiography. Front. Cardiovasc. Med..

[9] Becker M., Roehl A.B., Siekmann U., Koch A., de la Fuente M., Roissant R., Radermacher K., Marx N., Hein M. (2014). Simplified detection of myocardial ischemia by seismocardiography. Differentiation between causes of altered myocardial function. Herz.

[10] Inan O.T., Pouyan M.B., Javaid A.Q., Dowling S., Etemadi M., Dorier A., Heller J.A., Bicen A.O., Roy S., Marco T.D. (2018). Novel Wearable Seismocardiography and Machine Learning Algorithms Can Assess Clinical Status of Heart Failure Patients. Circ. Heart Fail..

[11] Iftikhar Z., Lahdenoja O., Tadi M.J., Hurnanen T., Vasankari T., Kiviniemi T., Airaksinen J., Koivisto T., Pänkäälä M. (2018). Multiclass classifier based cardiovascular condition detection using smartphone mechanocardiography. Sci. Rep..

[12] Hedén B., Öhlin H., Rittner R., Edenbrandt L. (1997). Acute myocardial infarction detected in the 12-lead ECG by artificial neural networks. Circulation.

[13] Haraldsson H., Edenbrandt L., Ohlsson M. (2004). Detecting acute myocardial infarction in the 12-lead ECG using Hermite expansions and neural networks. Artif. Intell. Med..

[14] Green M., Björk J., Forberg J., Ekelund U., Edenbrandt L., Ohlsson M. (2006). Comparison between neural networks and multiple logistic regression to predict acute coronary syndrome in the emergency room. Artif. Intell. Med..

[15] Olsson S.E., Ohlsson M., Ohlin H., Dzaferagic S., Nilsson M.L., Sandkull P., Edenbrandt L. (2006). Decision support for the initial triage of patients with acute coronary syndromes. Clin. Phys. Func. Imaging.

[16] Tripathy R.K., Bhattacharyya A., Pachori R.B. (2019). A novel approach for detection of myocardial infarction from ECG signals of multiple electrodes. IEEE Sens. J..

[17] Heuverswyn F.V., Buyzere M.D., Coeman M., Pooter J.D., Drieghe B., Duytschaever M., Gevaert S., Kayaert P., Vandekerckhove Y., Voet J. (2019). Feasibility and performance of a device for automatic self-detection of symptomatic acute coronary artery occlusion in outpatients with coronary artery disease: A multicentre observational study. Lancet Digit. Health.

[18] Spaccarotella C.A.M., Polimeni A., Migliarino S., Principe E., Curcio A., Mongiardo A., Sorrentino S., Rosa S.D., Indolfi C. (2020). Multichannel Electrocardiograms Obtained by a Smartwatch for the Diagnosis of ST-Segment Changes. JAMA Cardiol..

[19] Gibson C.M., Holmes D., Mikdadi G., Presser D., Wohns D., Yee M.K., Kaplan A., Ciuffo A., Eberly A.L., Iteld B. (2019). Implantable Cardiac Alert System for Early Recognition of ST-Segment Elevation Myocardial Infarction. J. Am. Coll. Cardiol..

[20] Myerburg R.J., Kessler K.M., Castellanos A. (1992). Sudden cardiac death: Structure, function, and time-dependence of risk. Circulation.

[21] Kannel W.B., Doyle J.T., McNamara P.M., Quickenton P., Gordon T. (1975). Precursors of sudden coronary death. Factors related to the incidence of sudden death. Circulation.

[22] Airaksinen K.E.J. (1999). Autonomic mechanisms and sudden death during abrupt coronary occlusion. Ann. Med..

[23] Kaplinsky E., Ogawa S., Balke C.W., Dreifus L.S. (1979). Two periods of early ventricular arrhythmia in the canine acute myocardial infarction model. Circulation.

[24] Naghavi M., Libby P., Falk E., Casscells S.W., Litovsky S., Rumberger J., Badimon J.J., Stefanadis C., Moreno P., Pasterkamp G. (2003). From Vulnerable Plaque to Vulnerable Patient: A Call for New Definitions and Risk Assessment Strategies: Part I. Circulation.

[25] Saczynski J.S., Yarzebski J., Lessard D., Spencer F.A., Gurwitz J.H., Gore J.M., Goldberg R.J. (2008). Trends in prehospital delay in patients with acute myocardial infarction (from the Worcester Heart Attack Study). Am. J. Cardiol..

[26] Inan O.T., Migeotte P.F., Park K.S., Etemadi M., Tavakolian K., Casanella R., Zanetti J., Tank J., Funtova I., Prisk G.K. (2015). Ballistocardiography and Seismocardiography: A Review of Recent Advances. IEEE J. Biomed. Health Inform..

[27] Shandhi M.M.H., Semiz B., Hersek S., Goller N., Ayazi F., Inan O.T. (2019). Performance Analysis of Gyroscope and Accelerometer Sensors for Seismocardiography-Based Wearable Pre-Ejection Period Estimation. IEEE J. Biomed. Health Inform..

[28] Tadi M.J., Mehrang S., Kaisti M., Lahdenoja O., Hurnanen T., Jaakkola J., Jaakkola S., Vasankari T., Kiviniemi T., Airaksinen J. (2019). Comprehensive Analysis of Cardiogenic Vibrations for Automated Detection of Atrial Fibrillation Using Smartphone Mechanocardiograms. IEEE Sens. J..

[29] Halvorsen P.S., Fleischer L.A., Espinoza A., Elle O.J., Hoff L., Skulstad H., Edvardsen T., Fosse E. (2009). Detection of myocardial ischaemia by epicardial accelerometers in the pig. Br. J. Anaesth..

[30] Theres H.P., Kaiser D.R., Nelson S.D., Glos M., Leuthold T., Baumann G., Sowelam S., Sheldon T.J., Stylos L. (2004). Detection of acute myocardial ischemia during percutaneous transluminal coronary angioplasty by endocardial acceleration. PACE-Pacing Clin. Electrophysiol..

[31] Harris I.S., Lee E., Yeghiazarians Y., Drew B.J., Michaels A.D. (2006). Phonocardiographic timing of third and fourth heart sounds during acute myocardial infarction. J. Electrocardiol..

[32] Siejko K.Z., Thakur P.H., Maile K., Patangay A., Olivari M. (2013). Feasibility of heart sounds measurements from an accelerometer within an icd pulse generator. PACE-Pacing Clin. Electrophysiol..

[33] Marcus G.M., Gerber I.L., McKeown B.H., Vessey J.C., Jordan M.V., Huddleston M., McCulloch C.E., Foster E., Chatterjee K., Michaels A.D. (2005). Association between phonocardiographic third and fourth heart sounds and objective measures of left ventricular function. J. Am. Med. Assoc..

[34] Morra S., Gauthey A., Hossein A., Rabineau J., Racape J., Gorlier D., Migeotte P., le Polain de Waroux J.B., van de Borne P. (2020). Influence of sympathetic activation on myocardial contractility measured with ballistocardiography and seismocardiography during sustained end-expiratory apnea. Am. J. Physiol.-Regul. Integr. Comp. Physiol..

